# Mental health outcomes in patients with a long-term condition: analysis of an Improving Access to Psychological Therapies service

**DOI:** 10.1192/bjo.2022.59

**Published:** 2022-06-01

**Authors:** Natasha Seaton, Rona Moss-Morris, Sam Norton, Katrin Hulme, Joanna Hudson

**Affiliations:** Institute of Psychology Psychiatry and Neuroscience, King's College London, UK; Institute of Psychology Psychiatry and Neuroscience, King's College London, UK; Institute of Psychology Psychiatry and Neuroscience, King's College London, UK; Institute of Psychology Psychiatry and Neuroscience, King's College London, UK; Institute of Psychology Psychiatry and Neuroscience, King's College London, UK

**Keywords:** comorbidity, depressive disorders, anxiety disorders, primary care

## Abstract

**Background:**

Having a long-term condition (LTC) significantly affects mental health. UK policy requires effective mental health provisions for patients with an LTC, generally provided by Improving Access to Psychological Therapies (IAPT) services. National IAPT data suggest that patients with an LTC typically demonstrate poorer outcomes compared with patients without an LTC. However, exploration of confounding factors and different outcome variables is limited.

**Aims:**

To establish the association of LTC status with demographic and clinical factors, and clinical mental health outcomes.

**Method:**

Anonymised patient-level data from a London IAPT service during January 2019 to October 2020 were used in this cohort study, to compare differences between LTC and non-LTC groups on sociodemographic and clinical variables. Binary logistic and multiple linear regression models were constructed for binary outcome variables (recovery and reliable improvement) and continuous outcomes (distress and functioning), respectively.

**Results:**

Patients with an LTC were more likely to be female; older; from a Black, mixed or other ethnic background; and have greater social deprivation. Across the four clinical outcomes (recovery, reliable improvement, final psychological distress and final functioning), having an LTC significantly predicted poorer outcomes even after controlling for sociodemographic and clinical baseline variables. For three outcome variables, greater social deprivation and being discharged during the COVID-19 pandemic also predicted poorer clinical outcomes.

**Conclusions:**

LTC status has a negative effect on mental health outcomes in IAPT services, independent of associated variables such as severity of baseline mental health symptoms, ethnicity and social deprivation. Effective psychological treatment for patients with an LTC remains an unresolved priority.

An estimated 30% of the UK population live with a chronic physical long-term condition (LTC).^1^ Physical LTCs elude conventional cures, but are managed with appropriate treatments, and include coronary heart disease, diabetes and chronic obstructive pulmonary disease. Patients diagnosed with an LTC experience higher rates of mental health problems, including anxiety and depression, compared with non-LTC populations.^2^ In LTCs, mental health issues are associated with poorer prognosis, increased risk of mortality and greater healthcare costs (45–75% increase).^1^ Qualitative data suggests that patients with an LTC struggle with the challenges brought on by diagnosis, are dissatisfied with their psychological care and find it hard to access relevant treatments.^3^ Improving Access to Psychological Therapies (IAPT) services are responsible for providing mental healthcare in England. From 2016, as part of the ‘NHS Five Year Forward View', patients with an LTC who are experiencing distress should be provided with access to integrated physical and mental healthcare.^4^ This resulted in IAPT publishing new LTC guidelines (IAPT-LTC) in 2018, to guide the implementation of integrated services.^5^ IAPT services monitor patient outcomes by asking them to complete brief measures of depression and anxiety at each appointment.^6^ IAPT define recovery as the proportion of patients whose scores on self-report measures of depression and anxiety are below clinical cut-offs used to indicate the presence of depression/anxiety at the end of treatment, having previously scored above these clinical cut-offs at the start of treatment.^7^

Since IAPT-LTC's implementation, recovery rates of 50% for depression and anxiety have been reported in patients with an LTC.^8^ This LTC recovery rate is in line with NHS Digital targets^9^ and observed rates in patients without an LTC.^5^ However, the three studies that we are aware of that statistically compare IAPT mental health outcomes for LTC and non-LTC populations all report poorer clinical outcomes for patients with an LTC.^10–12^ The three studies used data from before the implementation of IAPT-LTC guidelines and did not report recovery rates (IAPT's benchmark reporting criteria), thus preventing comparisons to nationally reported data. Moreover, two studies^11,12^ did not statistically control for key demographic confounders that increase the likelihood of having an LTC; namely, ethnicity^13^ and socioeconomic status.^14^ Ethnic minority groups and people of lower socioeconomic status are more likely to have poorer clinical outcomes in the UK.^15^ In IAPT specifically, some studies have shown that baseline depression and/or anxiety scores,^16^ ethnicity^10,15^ and socioeconomic status^10,15,16^ each exert effects on clinical outcomes. However, other studies find no relationship between clinical outcomes and either ethnicity^16,17^ or socioeconomic status.^17^ Conflicting findings may be explained by differences in the operationalisation of clinical outcomes used across studies. The differential operationalisation of mental health outcomes (e.g. dichotomous or continuous) obscures potential comparisons of studies with nationally reported data. An additional consideration is the increased mental health pressure exerted by the COVID-19 pandemic. Although the pandemic is increasing anxiety, depression and stress in the population,^18^ no studies to date have investigated IAPT data recorded during the COVID-19 pandemic.

In summary, the relationship between LTC status and clinical outcomes in IAPT are underexplored, particularly with regards to the potential confounding of demographic (gender, ethnicity, age, socioeconomic status) and baseline clinical measures. Investigations into IAPT services often examine only one clinical outcome, which renders comparisons between study results challenging.

## Aims

The aims of our study are to use routinely collected IAPT data to (a) establish the association of LTC status with demographic factors and clinical variables, such as baseline clinical scores and COVID-19 time frame; and (b) explore the association of LTC status with clinical outcomes, controlling for relevant demographic and clinical variables. Four outcomes were used in this study: a binary variable of recovery, a binary variable of reliable improvement, distress (Patient Health Questionnaire Anxiety and Depression Scale; PHQ-ADS) score and functioning (Work and Social Adjustment Scale; WSAS) score.

## Method

This paper is reported in accordance with the Strengthening the Reporting of Observational Studies in Epidemiology guidelines (for STROBE checklist, see Supplementary Material available at https://doi.org/10.1192/bjo.2022.59). The authors assert that all procedures contributing to this work comply with the ethical standards of the relevant national and institutional committees on human experimentation and with the Helsinki Declaration of 1975, as revised in 2008. This human study was approved by London NHS Quality Improvement Board (signed off by Director of Nursing at the relevant hospital trust on 7 January 2019). All adult participants provided verbal consent to participate because this is consistent with protocols in place for the national reporting of the IAPT services to NHS Digital. The verbal consent process was recorded/documented by healthcare professionals.

### Data

This study analysed available data, including raw demographic and clinical variables, collected from a large London IAPT service from 1 January 2019 to 20 October 2020, using any individual with a referral to the service during this period. All data analysed were collected as part of routine care for reporting to NHS Digital.^19^ This research was part of an NHS Quality Improvement project, approved by the Quality Improvement panel within the NHS Foundation trust that subsumes the IAPT service providing the anonymised data for the time period stated above. Anonymised data was used and therefore informed consent was not required. However verbal consent was provided by patients to their healthcare practitioner for their data to be shared with NHS Digital. This verbal consent process was recorded on clinical notes. Given that the researcher had no contact with the patients at the service, study bias was perceived to be low.

Participants were included if they had baseline outcomes from their initial assessment and at least one follow-up set of outcome scores for depression (Patient Health Questionnaire-9; PHQ-9^20^) and anxiety (seven-item Generalised Anxiety Disorder assessment; GAD-7^21^) for either a step 2 or step 3 level intervention. For patients with multiple follow-up outcomes, the last complete set was used in the analysis.

Out of the 20 650 referrals during the period, 13 762 participants were excluded because of incomplete baseline and/or outcome data. Participants were also excluded if there was no information recorded about their LTC status (*n* = 278), leaving 6610 participants. A subset of patients was excluded from the recovery analysis as they were below clinical cut-offs for both the PHQ-9 and GAD-7 at baseline (*n* = 802), and therefore data could not be used to classify as ‘recovered’ or ‘not recovered’. Where available, excluded and included participants were compared on demographic and baseline clinical factors. Excluded participants were more likely to be male, younger in age, and be from Black/Black British and mixed and other backgrounds. They had greater social deprivation and consistently higher baseline scores for depression, anxiety, psychological distress and impaired functioning (see Supplementary Table 1 for full results).

### Measures of clinical outcome

During initial assessment and at each appointment, IAPT collects three questionnaires: the PHQ-9, GAD-7 and the WSAS. The PHQ-9 measures depression.^20^ It has nine items, each scored on a four-point Likert scale. Scores range from 0 to 27 (a score ≥10 indicates clinically relevant symptoms). Higher scores indicate greater depression.^20^ The GAD-7 measures anxiety.^21^ It contains seven items scored on a four-point Likert scale. Total scores range from 0 to 21 (a score ≥8 indicates clinically relevant symptoms), with higher scores indicating greater anxiety.^21^ The WSAS measures functioning.^22^ It uses five items scored on a nine-point Likert scale. Scores range from 0 to 40, with higher scores indicating greater impairments in functioning.^22^

These questionnaires were used to construct the four primary outcomes in this study: recovery, reliable improvement, distress and functioning. Recovery (a binary outcome) is defined by IAPT as the patient improving in their self-report scores and also scoring below clinical cut-off points on self-report measures of depression and anxiety after treatment.^7^ Reliable improvement (a binary outcome) is defined by IAPT as patients demonstrating improvements in their self-reported scores on measures of depression and anxiety that are greater than the s.e. rates of that measure.^7^ Distress (a continuous outcome) was measured by the PHQ-ADS, which is calculated by summing the scores from the PHQ-9^20^ and GAD-7.^21^ It has a clinical cut-off of ≥10, indicating clinically significant psychological distress.^23^ The PHQ-ADS was selected for this study because it captures general psychological distress, which is thought to be more appropriate for patients with LTCs because of the coexistence of depression and anxiety in LTCs.^23^ Functioning (a continuous outcome) was assessed with the WSAS (see above).^22^

### Possible predictors of outcome

This study investigated eight potential predictors of outcome, all of which were derived from the routinely collected data in IAPT. The primary predictor of interest was LTC status. Other predictors included gender, age, ethnicity, social deprivation percentile and discharge date during the COVID-19 pandemic. Baseline PHQ-ADS and WSAS scores were used as clinical markers of distress severity. Ethnic categories were based on those reported by the Office of National Statistics and were as follows: White, Black or Black British, Asian or Asian British, mixed ethnicity and other. However, because of smaller numbers in the mixed ethnicity and other categories, these were combined, in line with other governmental reports on mental health among ethnic minorities.^15^

Based on the raw data, COVID-19 time frame and social deprivation percentile were computed before analysis. Patients’ postcodes were inputted into a publicly available government tool that gives deprivation data on the Lower Layer Super Output Area that each postcode falls under.^24^ Accordingly, the ranks were used to calculate the corresponding percentile for social deprivation, with a lower percentile indicating greater social deprivation. For the COVID-19 indicator, a discharge date before the beginning of the UK national lockdown on 23 March 2020 was coded as pre-COVID-19 pandemic. This ensured that any outcomes captured during the COVID-19 period would be included, despite referrals opening before the pandemic began.

### Statistical analyses

All statistical processes were performed in Stata (version 16, Windows). Because of the study's inclusion criteria and the IAPT monitoring system, there were very few cases of missing data. When data were missing, they were treated as blanks. The first aim addressed the differences in key demographic and clinical variables between those with an LTC and those without. These were calculated as either counts with percentages or means with s.d., depending on the level of measurement. *χ*^2^-Tests compared differences across categorical variables and *t*-tests compared continuous variables.

For the second aim, association between LTC status and key clinical outcomes were examined. Absolute association was examined with *χ*^2^-tests and individual sample *t*-tests for the binary and linear variables, respectively. For the period investigated, the official service recovery rate and recovery rates split by LTC status are reported with the same analytic criteria needed for reporting to NHS Digital. These criteria differ from those used in current study because patients are required to have completed a scheduled treatment and been formally discharged from the service to be eligible. This analysis aims to provide a comparison between this study's findings and nationally reported data.

Binary logistic regressions and linear regressions were used to determine the relative contribution of LTC status to the four outcome variables. Logistic regression models for recovery and reliable improvement reported odds ratios, *P*-values and 95% confidence intervals. For linear regressions, standardised and unstandardised beta values, *P*-values and 95% confidence intervals were reported. A 5% alpha level was applied for all tests. Variables were inputted in steps: the first step was LTC status. Next, key demographic variables were entered (gender, age, ethnicity, social deprivation), and finally, clinical variables (baseline scores and COVID-19 indicator) were inputted. To examine how the impact of LTCs varies in response to a person's sociodemographic profile; interaction effects between each demographic variable (gender, age, ethnicity, social deprivation) and LTC status were analysed in a fourth model for each outcome variable. For recovery, reliable improvement and final PHQ-ADS score, baseline PHQ-ADS score was controlled throughout all three steps to account for the imbalance at baseline between the LTC and non-LTC groups, and the intrinsic correlation between baseline and post-treatment outcomes. For the same reasons, the three steps of the WSAS linear regression had baseline WSAS scores controlled.

## Results

Table 1 shows the descriptive statistics and comparative analyses across the demographic and clinical variables between LTC and non-LTC groups. Across the 6610 participants, the mean age was 35.55 years (s.d. 12.67) and 68.77% were female. The participants were predominately (64.92%) from a White background and 31.39% identified as having an LTC. The average deprivation percentile was 0.344, indicating that the average individual was from a postcode in the 34th percentile, with the first percentile being the most socially deprived in the UK. All continuous variables (age, social deprivation, baseline clinical factors) were normally distributed.

**Table 1:** Demographic, clinical and process variables and their associations with long-term condition status

Variable	*n* (%)/mean (s.d.)	Non-LTC group, mean (s.d.)/%	LTC group, mean (s.d.)/%	Mean difference	Statistical test	*P*-value	95% CI
		*n* = 4535	*n* = 2075				
Demographic variables							
Gender							
Female	4542 (68.77%)	68.01%	70.43%		*χ*^2^ = 3.891	0.049*	
Male	2063 (31.23%)	31.99%	29.57%				
Ethnicity							
Asian or Asian British	447 (6.91%)	7.30%	6.06%		*χ*^2^ = 44.848	<0.001***	
Black or Black British	1133 (17.52%)	15.69%	21.53%				
Mixed and other	688 (10.64%)	10.01%	12.02%				
White	4198 (64.92%)	67.00%	60.39%				
Age, years	35.55 (12.67)	32.57 (10.32)	42.08 (14.73)	−9.514	*t* = −30.216	<0.001***	−10.1 to −8.9
Deprivation percentile	0.344 (0.18)	0.351 (0.18)	0.329 (0.17)	0.022	*t* = 4.499	<0.001***	−0.012 to −0.031
LTC status							
Present	2075 (31.39%)						
Absent	4535 (68.61%)						
Clinical variables							
Recovery							
Recovered	2153 (37.07%)	39.47%	31.95%		*χ*^2^ = 30.641	<0.001***	
Not recovered	3655 (62.93%)	60.53%	68.05%				
Reliable improvement							
Reliably improved	3488 (52.77%)	53.38%	51.42%		*χ*^2^ = 2.201	0.138	
No reliable improvement	3122 (47.23%)	46.26%	48.58%				
Baseline depression	13.77 (6.08)	13.18 (5.94)	15.06 (6.18)	−1.89	*t* = −11.830	<0.001***	−2.2 to −1.6
Baseline anxiety	12.70 (5.09)	12.37 (5.01)	13.43 (5.20)	−1.06	*t* = −7.869	<0.001***	−1.3 to −0.8
Baseline PHQ-ADS	26.47 (10.13)	25.54 (9.85)	28.49 (10.43)	−2.94	*t* = −11.06	<0.001***	−3.5 to −2.4
Baseline WSAS	18.42 (9.07)	17.60 (8.60)	20.22 (9.78)	−2.62	*t* = −10.995	<0.001***	−3.1 to −2.2
COVID-19 indicator					*χ*^2^ = 5.562	0.018*	
Discharged pre-COVID-19	2543 (38.47%)	39.43%	36.39%				
Discharged mid-COVID-19	4067 (61.53%)	60.57%	63.61%				
Final depression score (PHQ-9)	9.94 (6.42)	9.36 (6.16)	11.23 (6.79)	−1.86	*t* = −11.064	<0.001***	−2.2 to −1.5
Final anxiety score (GAD-7)	9.28 (5.75)	8.93 (5.61)	10.04 (5.98)	−1.11	*t* = −7.334	<0.001***	−1.4 to −0.8
Final PHQ-ADS score	19.22 (11.59)	18.29 (11.1)	21.3 (12.3)	−2.98	*t* = −9.769	<0.001***	−3.6 to −2.4
Final WSAS score	15.22 (9.57)	14.47 (9.1)	16.89 (10.4)	−2.42	*t* = −9.572	<0.001***	−2.9 to −1.9

LTC, long-term condition; PHQ-9, Patient Health Questionnaire-9; GAD-7, Generalised Anxiety Disorder-7 assessment; PHQ-ADS, Patient Health Questionnaire Anxiety-Depression Scale; WSAS, Work and Social Adjustment Scale.

* *P* < 0.05, **P < 0.01, ***P < 0.001.

### Differences between patients with and without an LTC

The first research aim was to investigate differences on demographic and clinical variables between patients with and without an LTC (inferential test data presented in Table 1). LTC status was positively associated with female gender (*P* = 0.049), age (*P* < 0.001) and deprivation percentile (*P* < 0.001). There was a significant association between ethnicity and LTC status (*P* < 0.001), with Black/Black British and mixed and other ethnicities having disproportionately greater numbers of patients with an LTC.

### Association of LTC status with primary outcomes

The second research aim addressed the association of LTC status with defined outcomes, statistically controlling for the other seven hypothesised explanatory predictor variables. Table 2 shows the odds ratio, *P*-value and 95% confidence interval of LTC status in each binary logistic regression model that controlled for potential explanatory variables. There were five cases of missing data: one case was missing PHQ-9 follow-up scores and four referrals were missing both GAD-7 and WSAS follow-up scores. Interaction effects between LTC status and sociodemographic predictors for each of the four outcomes are reported in the Supplementary Material.

**Table 2:** Odds ratios and standardised beta values for long-term condition status in regression models for the four outcomes

	Recovery			Reliable improvement			Final PHQ-ADS score				Final WSAS score			
	Odds ratio	*P-*value	95% CI	Odds ratio	*P-*value	95% CI	Standardised *β*	Unstandardised *β*	*P*-value	95% CI	Standardised *β*	Unstandardised *β*	*P*-value	95% CI
Model 1	0.874	0.032*	0.77 – 0.99	0.806	<0.001***	0.72 – 0.90	0.045	1.121	<0.001***	0.62–1.62	0.050	1.026	<0.001***	0.59–1.46
Model 2	0.839	0.010**	0.73 – 0.96	0.795	<0.001***	0.71 – 0.89	0.049	1.227	<0.001***	0.68–1.77	0.057	1.179	<0.001***	0.72–1.64
Model 3	0.857	0.025*	0.75 – 0.98	0.807	<0.001***	0.72 – 0.91	0.046	1.138	<0.001***	0.59–1.68	0.049	1.002	<0.001***	0.54–1.46

Variables included in model 1: LTC status, baseline PHQ-ADS score (for outcomes recovery, reliable improvement and PHQ-ADS) or baseline WSAS score (for WSAS outcome). Variables included in model 2: LTC status, gender, ethnicity, social deprivation percentile, age, baseline PHQ-ADS score (for outcomes recovery, reliable improvement and PHQ-ADS) or baseline WSAS score (for WSAS outcome). Variables included in model 3: LTC status, gender, ethnicity, social deprivation percentile, age, COVID-19 indicator, baseline PHQ-ADS score and baseline WSAS score. PHQ-ADS, Patient Health Questionnaire Anxiety-Depression Scale; WSAS, Work and Social Adjustment Scale; LTC, long-term condition.

* *P* < 0.05, **P < 0.01, ***P < 0.001.

### Recovery

Using the study's inclusion criteria, LTC status was negatively associated with recovery (*P* < 0.001), with only 31.95% of LTC referrals recovering compared with 39.47% of referrals without an LTC. However, when the data was analysed consistent with the protocols used for reporting outcomes to NHS Digital recovery analyses, the recovery rates for patients with and without an LTC was 45 and 56%, respectively, with the total sample showing a recovery rate of 51% over the time period investigated.

Across all three models, LTC status was negatively associated with recovery, with patients with an LTC 14.3% less likely (odds ratio 0.857) to recover than patients without a diagnosis, when demographic factors and clinical factors were controlled (*P* = 0.025) (see Table 2). Other significant positive predictors of recovery were older age (*P* = 0.014) and lower social deprivation (*P* = 0.012) (see Supplementary Table 2). Recovery was negatively predicted by higher baseline WSAS (*P* < 0.001) and PHQ-ADS (*P* < 0.001) scores and by being discharged within the COVID-19 time frame (*P* < 0.001), where patients were 28.7% less likely to recover if the patient was discharged after 23 March 2020. There were no statistically significant interaction effects between any of the demographic variables (gender, ethnicity, social deprivation or age) and LTC on recovery (see Supplementary Figs 1–4).

### Reliable improvement

All of the patients in the sample were eligible for the reliable improvement analysis, with 52.77% of referrals demonstrating a reliable improvement (See Table 1). There was no significant difference between LTC and non-LTC groups in a *χ*^2^-test (*P* = 0.138).

PHQ-ADS score was controlled throughout the three stages of the binary logistic regression models. LTC status was consistently a significant negative predictor of reliable recovery, with odds ratios ranging from 0.795 to 0.807 (see Table 2). In the third model, patients with an LTC were 19.3% less likely to reliably improve compared with those without an LTC (*P* < 0.0001). In the final binary logistic regression, lesser social deprivation was associated with greater reliable recovery (*P* = 0.040), whereas greater baseline clinical scores negatively predicted reliable improvement for both WSAS (*P* < 0.001) and PHQ-ADS (*P* < 0.001) scores (see Supplementary Table 3). There were no statistically significant interaction effects between any of the demographic variables (gender, ethnicity, social deprivation, age) and LTC on reliable recovery (see Supplementary Figs 5–8).

### Final PHQ-ADS score

LTC status was significantly associated with higher final PHQ-ADS score in an independent samples *t*-test, with *P <* 0.0001 (Table 1). Throughout the three linear regression models, LTC status significantly predicted higher final PHQ-ADS score (Table 2), with *P* < 0.001 in each model. In the third model, significant positive predictors of final PHQ-ADS score included the COVID-19 pandemic (*β* = 0.024, *P* = 0.020, 95% CI 0.09–1.05), baseline WSAS score (*β* = 0.097, *P* < 0.001, 95% CI 0.09–0.16) and baseline PHQ-ADS score (*β* = 0.488, *P* < 0.001, 95% CI 0.53–0.59). Final PHQ-ADS score was significantly negatively associated with lesser social deprivation (*β* = −0.032, *P* = 0.003, 95% CI −3.35 to −0.71) (Supplementary Table 4). Regarding interaction effects, the impact of the LTC was greater in female participants (*F*_1, 6438_ = 4.12, *P* = 0.042) when PHQ-ADS score was the outcome variable (see Supplementary Figs 9–12).

### Final WSAS score

LTC status was significantly associated with greater final impaired functioning on the WSAS scale (*P* < 0.0001) (Table 1). LTC status positively predicted greater final WSAS scores in all three models (Table 2). In the final model, greater final WSAS score was positively predicted by LTC status (*P* < 0.001), baseline WSAS score (*β* = 0.414, *P* < 0.001, 95% CI 0.41–0.46) and baseline PHQ-ADS score (*β* = 0.159, *P* < 0.001, 95% CI 0.13–0.17) (Supplementary Table 5). Interaction effects showed the negative impact of LTC on functioning was greater in older participants when WSAS was the outcome variable (*F*_1, 6413_ = 3.89, *P* = 0.049) (see Supplementary Figs 13–16).

## Discussion

### Summary of findings

This study used IAPT patient-level data from a large, real-world cohort to investigate the contribution of LTC status to clinical outcomes, controlling for demographic and baseline clinical factors. LTC status was negatively associated with recovery, with only 31.95% of patients with an LTC achieving recovery, compared with rates of 39.47% in patients without an LTC. These reported recovery rates are lower than those normally observed in the service, as the analysis included any patients with a pre- and post-treatment score, whereas standard recovery rates are calculated with patients who have completed treatment without dropping out. The association of LTC status with poorer recovery rates persisted when demographic and clinical factors were controlled, with patients with an LTC 14.3% less likely to recover. For the remaining three outcomes, regression analyses showed that patients with an LTC were less likely to demonstrate a reliable improvement and had significantly higher final PHQ-ADS and WSAS scores. These differences persisted after controlling for factors that predict poorer outcomes (including ethnicity, socioeconomic status and baseline clinical factors). Moreover, across the four clinical outcome measures, greater baseline outcome scores (depression, anxiety and impaired functioning) were significantly related to poorer outcomes. Greater social deprivation and being discharged during the COVID-19 pandemic were associated with poorer outcomes for recovery, reliable improvement and combined PHQ-ADS score. Interaction effects showed that the effect of having an LTC exacerbated final distress (PHQ-ADS) scores in females compared with males. Regarding age, in younger people, having an LTC makes little difference on final functioning scores; however, with increasing age, patients without an LTC demonstrated better final functioning scores than those with an LTC.

### Comparisons with previous research

Although past evidence corroborates poorer rates of reliable improvement^11^ and greater depression and anxiety severity post-treatment^10^ in patients with an LTC, this is the first study to show poorer clinical outcomes in this patient group across four key indicators, when demographics and baseline clinical scores are controlled. Although LTC status is associated with other factors that predispose poorer outcomes, such as ethnicity,^13^ lower socioeconomic status^14^ and higher baseline scores,^6,25^ this study shows that there is an additive effect of having an LTC, which makes recovery and improvement harder to achieve.

This study was conducted after the implementation of the IAPT-LTC initiative and highlights that there remains potential for increasing the effectiveness of IAPT-LTC interventions. Insufficient tailoring of interventions to LTC-specific challenges linked to the experience of depression and anxiety in LTCs, such as ongoing symptom management, challenging treatment regimens and illness uncertainty,^26^ may underly the poorer clinical outcomes observed in patients with an LTC. Often, conventional cognitive–behavioural therapy (CBT) protocols yield small effect sizes and show limited mental health and illness-related improvements among patients with an LTC.^27^ Insufficiently tailored protocols fail to address the relationship between mental health and chronic disease.^28^ Therefore, the omission of disease-related considerations might underly the reduced effectiveness of primary mental health protocols in patients with an LTC. CBT specifically adapted to LTC challenges yields better engagement, effectiveness and mental health outcomes in IAPT.^26^ Indeed, greater therapeutic relevance is associated with better engagement and lower levels of drop-out.^29^ In a randomised controlled trial, CBT tailored to diabetes showed larger improvements than standard CBT.^30^ Separately, psychological practitioners that received additional LTC training during a trial achieved higher recovery rates compared with those without tailored training,^31^ which is in accordance with an IAPT-wide study that found that higher numbers of highly trained staff were associated with better recovery rates.^6^ The IAPT-LTC implementation continues to train IAPT practitioners in LTC-specific competencies, thus this will likewise help to achieve better outcomes for patients with an LTC who are seeking treatment in IAPT services.^32^ However, a recent qualitative study, exploring IAPT therapists’ experiences of delivering treatments to patients with an LTC, cite system-level constraints as barriers to effective implementation; therefore, steps may need to be taken to address these systemic issues.^33^

Focusing on explanatory factors of poorer clinical outcomes other than LTCs, this study found poorer clinical outcomes during the COVID-19 pandemic, which reflects the higher rate of mental health problems documented since the beginning of the pandemic.^18^ Although lower recovery rates within IAPT services have been reported nationally,^19^ to our knowledge, this is the first study to establish COVID-19 as an independent predictor of poorer clinical outcomes, controlling for baseline mental health factors in IAPT services. National lockdowns have resulted in financial uncertainty and reduced quality of livelihood,^34^ which is likely to underly the substantial rise in number and severity of mental health concerns. Although higher baseline levels of depression and anxiety are generally associated with poorer recovery,^25^ our study suggests that the COVID-19 pandemic may have an additive negative impact on clinical outcomes. Systematic review evidence demonstrates the negative psychological effects of quarantine measures, and suggests that effects are sustained after the quarantine is lifted.^35^

Greater social deprivation was positively associated with having an LTC, and was a significant predictor of poorer clinical outcomes for recovery, reliable improvement and final PHQ-ADS score. This reflects prior evidence demonstrating the routine association of social deprivation with poorer mental health^36^ and poorer treatment outcomes.^37^ Interestingly, although there were disproportionately more patients with an LTC from either Black/Black British or mixed and other ethnic backgrounds, no ethnic group was a significant predictor of any of the clinical outcomes examined when LTC status and other demographic factors were controlled. This contrasts to findings from a past research study;^10^ however, the prior study did not control for socioeconomic status. The current study highlights the impact of social deprivation and the need for services and treatments to cater particularly to lower socioeconomic status groups.

### Limitations

There were nine factors that were associated with being excluded from the study: male gender; younger age; greater social deprivation; Black/Black British or mixed and other ethnic minority status; having an LTC; and higher baseline depression, anxiety, WSAS and PHQ-ADS scores. This is unlikely to confound our results as these factors were additionally associated with poorer outcomes, except for male gender and age. Individuals were primarily excluded because of failure to complete a second set of outcome measures (*n* = 9125), thus this finding may highlight the lack of acceptability of IAPT treatments for these individuals. It was beyond the scope of the current study to investigate causal factors contributing to premature discharge from IAPT; however, these findings indicate that a thorough investigation of predictive factors for disengagement (and underlying reasons) is required.

This study used data from a single London IAPT service, and therefore will be difficult to generalise to other UK regions and abroad, with different cultural factors and demographic compositions. However, appropriate comparisons can be made, as London is highly multicultural and the IAPT service used in this study ranks within the top 50 most deprived areas out of the 317 local authorities in England.^38^ Moreover, an arbitrary discharge date (23 March 2020) was used as the COVID-19 cut-off, so that any individuals discharged after the first UK lockdown had the COVID-19 time-frame classification. This definition excludes people who were affected by the global pandemic before the UK lockdown and fails to account for the degrees of restriction that varied temporally. However, since the sample sizes of the pre-COVID-19 and mid-COVID-19 groups were large, covering periods of at least 7 months, the approximation is sufficient to observe a reliable effect that has been reported nationally.^39^ Similar to other epidemiological studies, this study relied on self-reported LTC diagnoses. Furthermore, the data was absent regarding the specific LTC diagnosis, LTC severity, whether participants experienced distress related to their LTC and the number of attended therapy sessions.

In conclusion, this study has highlighted the impact of having an LTC on clinical outcomes in IAPT services, over and above a range of relevant factors, including severity of baseline mental health symptoms, ethnicity and social deprivation. This supports the need for targeted interventions to improve mental healthcare in this patient group. Given healthcare policy drives such as the NHS Five Year Forward View to improve integrated care for individuals with LTCs,^4^ the findings of this research highlight that mental healthcare treatment of patients with an LTC remains an unresolved priority. The study also suggests that a referral during the COVID-19 pandemic is associated with poorer clinical outcomes, even when clinical and demographic factors are controlled. Mental health services in the UK require increased support to cope with the increased difficulty of treating mental health concerns in the climate of the pandemic. Finally, there is a clinical need to develop a standardised measure of capturing whether distress is LTC-related (which would assist in improved triage) and provide tailored treatment for patients with LTCs.

## Data Availability

The data used in the study are available on request from the corresponding author, J.H. The data are not publicly available as they could compromise the privacy of the participants.

## Supplementary material

For supplementary material accompanying this paper visit http://doi.org/10.1192/bjo.2022.59. click here to view supplementary material
