# Supplementary material for: Mental health outcomes in patients with a long-term condition: analysis of an Improving Access to Psychological Therapies service
Source: BJPsych Open. 2022 Jun 1;8(4):e101. doi: 10.1192/bjo.2022.59 (PMC9230614; doi:10.1192/bjo.2022.59)
Supplement: Supplementary file 1 [file bjosup.zip › S205647242200059Xsup001.docx]

Supplementary Table 1. Demographic and clinical differences between included and excluded participants

| Variable | N (%) / M(SD) | Included group  M (SD) / % | Excluded group M(SD) /% | Mean difference | Statistical test | P value | 95%CI |
| --- | --- | --- | --- | --- | --- | --- | --- |
|  |  | n = 6,610 | n = 14,040 |  |  |  |  |
| **Demographic variables** |  |  |  |  |  |  |  |
| *Gender* |  |  |  |  |  |  |  |
| Female | 13,702 (66.45%) | 4,542 (68.77%) | 9,160 (65.36%) |  | χ^2^=23.318 | <0.001*** |  |
| Male | 6,917 (33.55%) | 2,063 (31.23%) | 4,854 (34.64%) |  |  |  |  |
| *Ethnicity* |  |  |  |  |  |  |  |
| Asian or Asian British | 1,223 (6.51%) | 447 (6.91%) | 776 (6.30%) |  | χ^2^=75.581 | <0.001*** |  |
| Black or Black British | 3,842 (20.45%) | 1,133 (17.52%) | 2,709 (21.99%) |  |  |  |  |
| Mixed & Other | 2,208 (11.75%) | 688 (10.64%) | 1,520 (12.34%) |  |  |  |  |
| White | 11,512 (61.28%) | 4,198 (64.92%) | 7,314 (59.37%) |  |  |  |  |
| Age | 35.31 (13.20) | 35.55 (12.67) | 35.20 (13.45) | -0.35 | t=-1.78 | *p*=0.0374* | -.74 – -.04 |
| Deprivation percentile | 0.331 (0.17) | 0.344 (0.18) | 0.325 (0.17) | -0.019 | t=-7.368 | <0.001*** | -.024 – -.014 |
| **Clinical variables** |  |  |  |  |  |  |  |
| Baseline depression | 14.28 (6.31) | 13.77 (6.08) | 14.76 (6.47) | 0.99 | t=9.318 | <0.001*** | 0.79 – 1.20 |
| Baseline anxiety | 12.84 (5.26) | 12.70 (5.09) | 12.99 (5.40) | 0.27 | t=3.012 | <0.001*** | 0.09 – 0.44 |
| Baseline PHQ-ADS | 27.13 (10.61) | 26.47 (10.13) | 27.73 (10.99) | 1.27 | t=7.034 | <0.001*** | 0.91 – 1.62 |
| Baseline WSAS | 19.05 (9.59) | 18.42 (9.07) | 19.63 (10.01) | 1.20 | t=7.383 | <0.001*** | 0.88 – 1.52 |

Supplementary Table 2. Binary logistic regression predicting recovery with variables entered in three stages

| Variable | | OR | p value | 95% CI | OR | p value | 95% CI | OR | p value | 95% CI |
| --- | --- | --- | --- | --- | --- | --- | --- | --- | --- | --- |
| LTC | | 0.874 | 0.032 | .77 – .99 | 0.839 | 0.010 | .73 – .96 | 0.857 | 0.025 | .75 – .98 |
| Gender (Female) | |  |  |  | 0.952 | 0.436 | .84 – 1.08 | 0.937 | 0.307 | .83 – 1.06 |
| Ethnicity (White) | |  |  |  |  |  |  |  |  |  |
|  | Asian or Asian British |  |  |  | 0.895 | 0.339 | .71 – 1.12 | 0.909 | 0.411 | .72 – 1.14 |
|  | Black or Black British |  |  |  | 1.044 | 0.581 | .89 – 1.22 | 1.091 | 0.277 | .93 – 1.28 |
|  | Mixed & Other |  |  |  | 0.868 | 0.339 | .72 – 1.05 | 0.884 | 0.212 | .73 – 1.07 |
| Social deprivation percentile | |  |  |  | 1.520 | 0.010 | 1.10 –2.09 | 1.511 | 0.012 | 1.10 –2.08 |
| Age | |  |  |  | 1.005 | 0.036 | 1.00 –1.01 | 1.006 | 0.014 | 1.00 –1.01 |
| COVID-19 | |  |  |  |  |  |  | 0.713 | <0.001 | .63 – .80 |
| Baseline WSAS | |  |  |  |  |  |  | 0.981 | <0.001 | .97 – .99 |
| Baseline PHQ-ADS | | 0.921 | <0.001 | .91 – .93 | 0.922 | <0.001 | .91 – .93 | 0.931 | <0.001 | .92 – .94 |

** denotes p value ≤0.05; ** denotes p value ≤0.01; *** denotes p value ≤0.001*

*PHQ-ADS, Patient Health Questionnaire Anxiety-Depression Scale; WSAS, Work and Social Adjustment Scale*

| Supplementary Figure 1. Interaction effects of LTC and gender on recovery |  | Supplementary Figure 2. Interaction effects of LTC and ethnicity on recovery |
| --- | --- | --- |
|  |  |  |
| Supplementary Figure 3. Interaction effects of LTC and social deprivation on recovery |  | Supplementary Figure 4. Interaction effects of LTC and age on recovery |
|  |  |  |

Supplementary Table 3. Binary logistic regression predicting reliable change with variables entered in three stages

| Variable | | OR | p value | 95% CI | OR | p value | 95% CI | OR | p value | 95% CI |
| --- | --- | --- | --- | --- | --- | --- | --- | --- | --- | --- |
| LTC | | 0.806 | <0.001*** | .72 – .90 | 0.795 | <0.001*** | .71 – .89 | 0.807 | <0.001*** | .72 – .91 |
| Gender (Female) | |  |  |  | 1.023 | 0.679 | .92 – 1.14 | 1.001 | 0.989 | .90 – 1.12 |
| Ethnicity (White) | |  |  |  |  |  |  |  |  |  |
|  | Asian or Asian British |  |  |  | 1.019 | 0.854 | .83 – 1.25 | 1.031 | 0.768 | .84 – 1.26 |
|  | Black or Black British |  |  |  | 0.988 | 0.868 | .86 – 1.13 | 1.022 | 0.763 | .89 – 1.17 |
|  | Mixed & Other |  |  |  | 0.919 | 0.319 | .78 – 1.09 | 0.933 | 0.418 | .79 – 1.10 |
| Social deprivation percentile | |  |  |  | 1.372 | 0.029* | 1.0 –1.82 | 1.014 | 0.040* | 1.01 –1.80 |
| Age | |  |  |  | 1.001 | 0.568 | 1.00–1.01 | 0.998 | 0.348 | 1.00 –1.01 |
| COVID-19 | |  |  |  |  |  |  | 0.713 | <0.001*** | .84 –1.03 |
| Baseline WSAS | |  |  |  |  |  |  | 0.981 | <0.001*** | .97 – .98 |
| Baseline PHQ-ADS | | 1.046 | <0.001*** | 1.04– 1.05 | 1.048 | <0.001*** | 1.04–1.05 | 0.931 | <0.001*** | 1.05 – 1.07 |

** denotes p value ≤0.05; ** denotes p value ≤0.01; *** denotes p value ≤0.001*

*PHQ-ADS, Patient Health Questionnaire Anxiety-Depression Scale; WSAS, Work and Social Adjustment Scale*

| Supplementary Figure 5. Interaction effects of LTC and gender on reliable improvement |  | Supplementary Figure 6. Interaction effects of LTC and ethnicity on reliable improvement |
| --- | --- | --- |
|  |  |  |
| Supplementary Figure 7. Interaction effects of LTC and social deprivation on reliable improvement |  | Supplementary Figure 8. Interaction effects of LTC and age on reliable improvement |
|  |  |  |

Supplementary Table 4. Linear regression predicting final PHQ-ADS score with variables entered in three stages

| Variable | | Coef | Standardised β | p value | 95% CI | Coef | Standardised β | p value | 95% CI | Coef | Standardised β | p value | 95% CI |
| --- | --- | --- | --- | --- | --- | --- | --- | --- | --- | --- | --- | --- | --- |
| LTC | | 1.121 | 0.045 | <0.001*** | .62 – 1.62 | 1.227 | 0.049 | <0.001*** | .68 – 1.77 | 1.138 | 0.046 | <0.001*** | .59 – 1.68 |
| Gender (female) | |  |  |  |  | 0.199 | 0.008 | 0.443 | -.31 - .71 | 0.320 | 0.013 | 0.244 | -.29 - .83 |
| Ethnicity | |  |  |  |  |  |  |  |  |  |  |  |  |
|  | Asian or Asian British |  |  |  |  | 0.072 | 0.002 | 0.880 | -.87–1.01 | -0.002 | > -0.001 | 0.996 | -.94 – .94 |
|  | Black or Black British |  |  |  |  | -0.542 | -0.002 | 0.869 | -.70 – .59 | -0.240 | -0.008 | 0.464 | -.88 – .40 |
|  | Mixed & Other |  |  |  |  | 0.514 | 0.014 | 0.196 | -.27 – 1.29 | 0.432 | 0.012 | 0.275 | -.34 – 1.21 |
| Social deprivation percentile | |  |  |  |  | -2.123 | -0.033 | 0.002** | -3.45 – -.80 | -2.032 | -0.032 | 0.003** | -3.35 – -.71 |
| Age | |  |  |  |  | -0.010 | -0.011 | 0.307 | -.03 – .01 | -0.015 | -0.016 | 0.144 | -.03 - .01 |
| COVID-19 | |  |  |  |  |  |  |  |  | 2.024 | 0.024 | 0.020* | .09 – 1.05 |
| Baseline WSAS | |  |  |  |  |  |  |  |  | 0.129 | 0.097 | <0.001*** | .09 - .16 |
| Baseline PHQ-ADS | | 0.631 | 0.552 | <0.001*** | .61 – .65 | 0.624 | 0.546 | <0.001*** | .60 - .65 | 0.531 | 0.488 | <0.001*** | .53 - .59 |

** denotes p value ≤0.05; ** denotes p value ≤0.01; *** denotes p value ≤0.001*

*PHQ-ADS, Patient Health Questionnaire Anxiety-Depression Scale; WSAS, Work and Social Adjustment Scale*

| Supplementary Figure 9. Interaction effects of LTC and gender on final PHQ-ADS |  | Supplementary Figure 10. Interaction effects of LTC and ethnicity on final PHQ-ADS |
| --- | --- | --- |
|  |  |  |
| Supplementary Figure 11. Interaction effects of LTC and social deprivation on final PHQ-ADS |  | Supplementary Figure 12. Interaction effects of LTC and age on final PHQ-ADS |
|  |  |  |

Supplementary Table 5. Linear regression predicting final WSAS score with variables entered in three stages

| Variable | | Coef | Standardised β | p value | 95% CI | Coef | Standardised β | p value | 95% CI | Coef | Standardised β | p value | 95% CI |
| --- | --- | --- | --- | --- | --- | --- | --- | --- | --- | --- | --- | --- | --- |
| LTC | | 1.026 | 0.050 | <0.001*** | .59 – 1.46 | 1.179 | 0.057 | <0.001*** | .72 – 1.64 | 1.002 | 0.049 | <0.001*** | .54 – 1.46 |
| Gender (female) | |  |  |  |  | 0.049 | 0.002 | 0.825 | -.39 - .48 | -0.125 | -0.006 | 0.569 | -.56 - .31 |
| Ethnicity | |  |  |  |  |  |  |  |  |  |  |  |  |
|  | Asian or Asian British |  |  |  |  | 0.434 | 0.012 | 0.288 | -.37–1.23 | 0.256 | 0.007 | 0.526 | -.54–1.05 |
|  | Black or Black British |  |  |  |  | 0.053 | 0.002 | 0.852 | -.50 – .60 | -0.059 | -0.002 | 0.833 | -.60 – .49 |
|  | Mixed & Other |  |  |  |  | 0.193 | 0.006 | 0.569 | -.47 – .86 | 0.056 | 0.002 | 0.868 | -.60 – .71 |
| Social deprivation percentile | |  |  |  |  | -1.359 | -0.026 | 0.018* | -2.49 – -.23 | -1.009 | -0.019 | 0.077 | -2.13 – .11 |
| Age | |  |  |  |  | -0.013 | -0.017 | 0.130 | -.03 – .00 | -0.012 | -0.016 | 0.168 | -.03 – .01 |
| COVID-19 | |  |  |  |  |  |  |  |  | 0.026 | 0.001 | 0.899 | -.38 – .43 |
| Baseline PHQ-ADS | |  |  |  |  |  |  |  |  | 0.150 | 0.159 | <0.001*** | .13 - .17 |
| Baseline WSAS | | 0.534 | 0.506 | <0.001*** | .51 – .56 | 0.534 | 0.508 | <0.001*** | .51 - .56 | 0.436 | 0.414 | <0.001*** | .41 - .46 |

** denotes p value ≤0.05; ** denotes p value ≤0.01; *** denotes p value ≤0.001*

*PHQ-ADS, Patient Health Questionnaire Anxiety-Depression Scale; WSAS, Work and Social Adjustment Scale*

| Supplementary Figure 13. Interaction effects of LTC and gender on final WSAS |  | Supplementary Figure 14. Interaction effects of LTC and ethnicity on final WSAS |
| --- | --- | --- |
|  |  |  |
| Supplementary Figure 15. Interaction effects of LTC and social deprivation on final WSAS |  | Supplementary Figure 16. Interaction effects of LTC and age on final WSAS |
|  |  |  |
