# Supplementary material for: Mental health outcomes in patients with a long-term condition: analysis of an Improving Access to Psychological Therapies service
Source: BJPsych Open. 2022 Jun 1;8(4):e101. doi: 10.1192/bjo.2022.59 (PMC9230614; doi:10.1192/bjo.2022.59)
Supplement: Supplementary file 1 [file bjosup.zip › S205647242200059Xsup002.docx]

Appendix 1 – STROBE checklist

| Tick |  | Item No | Recommendation |
| --- | --- | --- | --- |
|  | **Title and abstract** | 1 | (*a*) Indicate the study’s design with a commonly used term in the title or the abstract |
|  |  |  | (*b*) Provide in the abstract an informative and balanced summary of what was done and what was found |
|  | Introduction | | |
|  | Background/rationale | 2 | Explain the scientific background and rationale for the investigation being reported |
|  | Objectives | 3 | State specific objectives, including any prespecified hypotheses |
|  | Methods | | |
|  | Study design | 4 | Present key elements of study design early in the paper |
|  | Setting | 5 | Describe the setting, locations, and relevant dates, including periods of recruitment, exposure, follow-up, and data collection |
|  | Participants | 6 | (*a*) Give the eligibility criteria, and the sources and methods of selection of participants. Describe methods of follow-up |
| NA |  |  | (*b*) For matched studies, give matching criteria and number of exposed and unexposed |
|  | Variables | 7 | Clearly define all outcomes, exposures, predictors, potential confounders, and effect modifiers. Give diagnostic criteria, if applicable |
|  | Data sources/ measurement | 8* | For each variable of interest, give sources of data and details of methods of assessment (measurement). Describe comparability of assessment methods if there is more than one group |
|  | Bias | 9 | Describe any efforts to address potential sources of bias |
|  | Study size | 10 | Explain how the study size was arrived at |
|  | Quantitative variables | 11 | Explain how quantitative variables were handled in the analyses. If applicable, describe which groupings were chosen and why |
|  | Statistical methods | 12 | (*a*) Describe all statistical methods, including those used to control for confounding |
|  |  |  | (*b*) Describe any methods used to examine subgroups and interactions |
|  |  |  | (*c*) Explain how missing data were addressed |
| NA |  |  | (*d*) If applicable, explain how loss to follow-up was addressed |
| NA |  |  | (*e*) Describe any sensitivity analyses |
|  | Results | | |
|  | Participants | 13* | (a) Report numbers of individuals at each stage of study—eg numbers potentially eligible, examined for eligibility, confirmed eligible, included in the study, completing follow-up, and analysed |
|  |  |  | (b) Give reasons for non-participation at each stage |
| NA |  |  | (c) Consider use of a flow diagram |
|  | Descriptive data | 14* | (a) Give characteristics of study participants (eg demographic, clinical, social) and information on exposures and potential confounders |
| NA |  |  | (b) Indicate number of participants with missing data for each variable of interest |
|  |  |  | (c) Summarise follow-up time (eg, average and total amount) |
|  | Outcome data | 15* | Report numbers of outcome events or summary measures over time |
|  | Main results | 16 | (*a*) Give unadjusted estimates and, if applicable, confounder-adjusted estimates and their precision (eg, 95% confidence interval). Make clear which confounders were adjusted for and why they were included |
| NA |  |  | (*b*) Report category boundaries when continuous variables were categorized |
|  |  |  | (*c*) If relevant, consider translating estimates of relative risk into absolute risk for a meaningful time period |
| NA | Other analyses | 17 | Report other analyses done—eg analyses of subgroups and interactions, and sensitivity analyses |
|  | Discussion | | |
|  | Key results | 18 | Summarise key results with reference to study objectives |
|  | Limitations | 19 | Discuss limitations of the study, taking into account sources of potential bias or imprecision. Discuss both direction and magnitude of any potential bias |
|  | Interpretation | 20 | Give a cautious overall interpretation of results considering objectives, limitations, multiplicity of analyses, results from similar studies, and other relevant evidence |
|  | Generalisability | 21 | Discuss the generalisability (external validity) of the study results |
|  | Other information | | |
|  | Funding | 22 | Give the source of funding and the role of the funders for the present study and, if applicable, for the original study on which the present article is based |

*Information given separately for exposed (LTC present) and unexposed (LTC absent) groups.
